# Utilization, Determinants, and Prospects of Electronic Medical Records in Ethiopia

**DOI:** 10.1155/2021/2230618

**Published:** 2021-11-08

**Authors:** Abdu Oumer, Ahmed Muhye, Imam Dagne, Nesredin Ishak, Ahmed Ale, Abiyot Bekele

**Affiliations:** ^1^Department of Public Health, College of Medicine and Health Sciences, Dire Dawa University, Dire Dawa, Ethiopia; ^2^School of Public Health, College of Medicine and Health Sciences, Bahir Dar University, Ethiopia; ^3^Cluster Coordinator and Deliverology & HHS Advisor, Engender Health at Harari Regional Health Bureau, Harar, Ethiopia; ^4^School of Medicine, College of Medicine and Health Sciences, Dire Dawa University, Dire Dawa, Ethiopia; ^5^Health Monitoring and Evaluation Senior Expert, Dire Dawa Administration Health Bureau, Dire Dawa, Ethiopia

## Abstract

**Background:**

A lot of effort is being done in the electronic medical record (EMR) system. However, it has not been implemented and used at the expected scale for maximal effectiveness. There is limited evidence on the factors affecting the utilization of EMR in this particular context, which are critical for targeted strategies.

**Objective:**

To assess the magnitude and factors affecting the utilization of EMR among health professionals in eastern Ethiopia.

**Methods:**

An institutional-based cross-sectional study was conducted among randomly selected 412 health professionals from Harari and Dire Dawa, eastern Ethiopia, using a pretested self-administered questionnaire. The tool was developed from previous literature, and a pilot survey was done before the actual study. Bivariable and multivariable binary logistic regression were done to assess the relationship between an independent variable with EMR use. Crude and an adjusted odds ratio with a 95% confidence interval were reported. A *P* value of less than 0.05 was used to declare a statistically significant association.

**Results:**

A total of 412 health professionals with a mean age of 29 years (±6.4 years) were included. A total of 229 (55.6%) and 300 (72.8%) of them had good knowledge and attitude towards the EMR, while 279 (67.7%) used the service (54% used it on a daily basis). About 272 (66%) of the respondents reported that they prefer EMRs to paper-based systems. Health professionals with more than five years of experience had two times higher odds of using the service (AOR = 2.22; 95% CI; 1.12-4.42) than early-career workers. Health professionals trained in EMR would use the service more (AOR = 5.88; 95% CI; 2.93-11.88) compared to those who did not take the training. In addition, having good knowledge (AOR = 1.52; 95% CI; 0.92-1.5) and a good attitude towards the EMR system (AOR = 2.4; 95% CI; 1.35-4.31) showed to use EMR as compared to counterparts.

**Conclusions:**

The utilization of EMR was found to be optimal. Age, work experience, knowledge, attitude, and training of professionals were positively associated with the use of the service in their facility.

## 1. Introduction

Health records are the most important database of the patient record, which consists of various data entered by health care professionals in either paper or electronic form [1–4]. A computer-based record (CPR) is a depository of electronically maintained information about an individual's lifetime health status and health care which has the objective of supporting patient care and improving the quality of health care [5]. This is all about saving a life by facilitating communication, practicing evidence-based decisions, and many others [6, 7]. Among recent information technology (IT) system initiatives in developing countries, EMR systems are becoming dominant with the vision of improving data handling and communication in healthcare organizations [8, 9]. The development of the EMR system helped institutions to handle patient records in a wise, safe, and qualified manner. However, these systems have various problems and barriers that can hinder their implementation at a better scale or ideal scope [3, 7, 10–12].

EMR is computerized medical information systems that collect, store, and display patient information. It can include a wide range of information, including sociodemographics, insurance, medications, intake history, allergies, laboratory test results, immunization, hospitalization history, and others, all while maintaining patient privacy and confidentiality [7, 13]. They are a means of creating legible and organized recordings and of accessing clinical information about individual patients [7, 14].

It has been suggested that wide-scale adoption and implementation of EMRs could be pivotal for improving patient safety and health care quality [15–17]. It may also reduce the costs of providing ambulatory care. However, despite emerging evidence about the benefits of EMRs, there are considerable barriers to their adoption [14, 18]. This urges the need for developing countries to have evidence-based improvement and scale-up of EMR systems within their facilities [19].

However, low-income countries have struggled to launch large-scale EMR systems in their context, owing to a lack of resources as well as the necessary skills and infrastructure. These systems require abundant resources including skilled labor, technological, and financial means, all of which can be difficult to procure in low-income settings [20–22].

Despite the high expectations and interest in adopting and using the EMR systems, its overall adoption is relatively low, especially in resource-limited countries where high disease prevalence and incidence rates are predominant [13, 14, 18, 23, 24]. As indicated by various studies, the adoption and use of EMR systems in developing countries is still in its embryonic stage for several reasons [13, 23]. Users' attitudes, knowledge, technical skills, the functionality of the working environment/infrastructure, and lack of adequate resources are pointed out as important determinants for the functionality of the adopted EMR system [25].

The utilization of EMR can be affected by different factors. A complex span of factors determines the adoption and use of EMR by health professionals despite established and functional EMR in place [25]. Health professionals' attitude and awareness level, lack of proper management, resource shortage, skill-related issues, users' resistance, policy-related issues, poor commitments of staff, and poor maintenance services are other reasons for the limited adoption and use of EMR systems in developing countries [26–29].

In Ethiopia, the organization, availability, accessibility, and quality of health data are still poor. Moreover, information is not being stored and used effectively in health care, resulting in inappropriate and uncertain clinical decision-making [30, 31]. The Ethiopian government is training health informatics professionals to support the health management information system and transform the health care system [25]. Studies show that the adoption of an EMR system in the healthcare system has the potential to transform healthcare in terms of saving costs, reducing medical errors, improving service quality, increasing patients' safety, decision-making, saving time, data confidentiality, and sharing medical information [24, 32–34].

So far, limited studies have been conducted to give an insight in to EMR acceptance, use, and other issues in some parts of the country where the EMR implementation and scale of adoption are different [30, 35, 36]. The utilization rate was 71% [30] in the northern part of Ethiopia and 33% in India [35]. Higher levels of education, training on EMR, management support, and computer literacy have been shown to be associated with EMR use [30, 35, 36], but with inconsistent evidence. Various health facilities in developing countries are using EMR systems in varying degrees for several reasons [25]. Eastern Ethiopia is one of the areas, where a large proportion of people reside with different disease patterns. Governments' need to deliver evidence-based health services was the basic reason for adopting EMR systems in developing countries [34, 36, 37]. This urges the need to find context-specific evidence on the level of adoption, EMR implementation utilization, and barriers for not using the system. This particular research tried to pinpoint the magnitude of the utilization of EMR and its associated factors among health professionals in governmental health facilities in Eastern Ethiopia.

## 2. Method and materials

### 2.1. Study setting

This study was conducted in Eastern Ethiopia (Dire Dawa, Eastern Harerghe, Harar, and Ethiopian Somali). Thus, out of these study sites, three areas reported to have an established EMR system in their health care system, namely, the Dire Dawa Administration, the Harari Regional State, and Ethiopian Somali. Harar is located 526 km east of Addis Ababa, the capital city of Ethiopia. The two regions and the administrative town together comprise more than 4.5 million population. All are located in the eastern part of the country. There are about 6755 health care workers working in these regions, including the Harari region [38].

### 2.2. Study design

This is an institutional-based quantitative cross-sectional study that was conducted to assess EMR utilization and its associated factors among health professionals in eastern Ethiopia.

### 2.3. Population

Voluntary health professionals who are working in health facilities in Eastern Ethiopia where there is a functional EMR system within the facility. All randomly selected health professionals from all categories working in the selected health facilities where there is a functional EMR system within the facilities were included in the study. While those who were on annual or maternal leave were not included in this study.

### 2.4. Sample size determination

The sample size was determined using a single population proportion formula at 95% confidence level, 5% significance level, EMR utilization among health care workers (p) of 70.8% [30], a desired degree of precision (d) of 5%, and a design effect of 1.5. The sample size for factors associated with EMR utilization was calculated using sample size calculation for double proportion (under Epi info version 7.0 software for sample size and power calculation) by taking power (80%), 95% confidence level, and utilization estimates from previous studies. The final sample size became 525 with the inclusion of a 5% nonresponse rate. However, because the total number of eligible health professionals working in a facility where EMR service was not available was greater than the number of eligible study participants, all eligible study participants were included.

### 2.5. Sampling procedure/techniques

A stratified sampling technique with proportionate allocation to each region and health facility (sample size proportional to size) was employed. First, the total sample size was stratified into two regions where the service is functional. Then, further stratification by type of health facility was done to hospitals and health centers where the service is functional. Thus, health professionals working in facilities without functional EMR were not considered.

**Figure 1:**
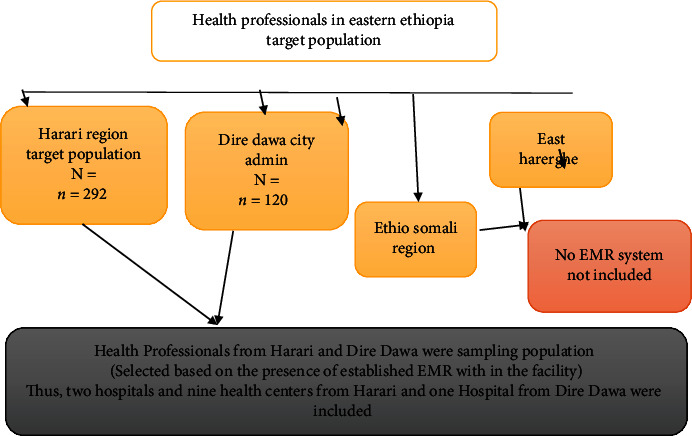
Diagrammatic summary of the sample size (sampling procedure) for each region and city administration based on the stratification and proportion of their health care work force.

The study samples were proportionally allocated to each health facility depending on the number of health professionals within that facility. To develop a sampling frame, the list of health facilities and health care workers was obtained from the health bureau of the respective regions and city administration. However, as the EMR system is available in two sites (namely, Harar and Dire Dawa one health facility only), the sampling population became smaller and all available health professionals working on all facilities with established EMR systems were included (Figure 1).

### 2.6. Data collection instrument and technique

A self-administered structured questionnaire was used to collect data on sociodemographic, organizational, and technology-related factors, as well as knowledge, attitude, and use of electronic medical records. The questionnaire was adopted from previous studies [17, 37, 39–41]. The questionnaire was prepared in the English language. Regarding data collection, diploma health informatics students and technicians were involved in administering the questionnaire after they took two days of training. Bachelor degree (BSC) holders from any health science field worked closely with investigators to oversee the data collection process.

### 2.7. Data quality issues

Structured self-administered questionnaires were adopted from previous studies and checked for consistency. The data collection information sheet was developed by the investigator on the objective of the study, how to collect data (technique of data collection), ethical issues, and a description of inclusion and exclusion criteria, and training was provided for the data collectors. All filled-out questionnaires were reviewed by the data collectors for clarity, completeness, and relevance. Close supervision was done accordingly. The collected data was entered in a prespecified format into Epi Data version 3.01, for consistency, double data entry, restricting entry through legal values, and skipping patterns.

### 2.8. Variables of the study

The dependent variable of this study was the utilization of EMR (utilized or not utilized), while sociodemographic variables (age, sex, income, educational level, and professional category), years of service, technology-related variables, access to computer, knowledge, attitudes, and training on EMR were independent variables considered.

### 2.9. Data processing and analysis

Data were entered into Epi Data version 3.01 and cleaned and analyzed using SPSS version 20 statistical software. Descriptive analyses such as frequency, percentages, graphic presentations, and summary tables were conducted for categorical variables. Bivariate logistic regression was performed for each independent variable against the outcome variable (EMR utilization) to estimate the crude odds ratio. The main purposes of EMR utilization for data recording, storing, retrieving, reporting, and other eight core functions of EMR in a daily task were considered in assessing the EMR's utilization by health professionals. Thus, those with reported use of EMR for the stated purposes were categorized as EMR system users, whereas those who did not use the EMR for the abovementioned (twelve core functions) tasks were considered as nonusers of the EMR system. Health professionals' knowledge of the EMR system was assessed using a set of questions adapted from previous literature, and the sum score was calculated. Based on the median of the sum knowledge score (skewed distribution), those who scored greater than or equal to the median score were categorized as having good knowledge of EMR. Similarly, an attitude score was generated, and the median attitude sum score was used to classify individuals as having a good or poor attitude towards the EMR system, respectively.

A stepwise backward binary logistic regression was used to identify factors associated with the utilization of EMR. Both bivariate and multivariate binary logistic regressions were used. Predictor variables associated with outcome at a *P* value below 0.2 and important predictor variables identified in previous literature were considered for the multivariable analysis. The multivariable binary logistic regression method was used to assess the factors associated with the utilization of EMR with each identified predictor variable. An adjusted odds ratio (AOR) with a *P* value and a 95% confidence interval was reported. Associations with a *P* value below 0.05% in multivariate analysis were declared as statistically significant predictors of EMR utilization among health professionals.

The goodness of fit of the model was assessed using Hosmer-Lemeshow's statistical test with a *P* value above 0.5 as a fitted logistic regression model. In addition, a significant omnibus test and improved classification precision were also assessed for model specification.

### 2.10. Ethical considerations

Ethical approval was obtained from the research and technology interchange (RTI) of Dire Dawa University (DDU), and a support letter was taken to each region and facility for official communications. Verbal informed consent was obtained from each health professional after a detailed explanation of the purpose, confidentiality, benefits, risks, and procedures during data collection. Privacy and confidentiality were maintained by not asking for personal identifiers like names and addresses. The respondent's anonymity to withdraw from the study during the course of data collection was maintained. Personal identity identifiers were not collected.

## 3. Results

### 3.1. Characteristics of study participants

A total of 412 health professionals from different categories and educational levels with a mean age of 29 years (±6.4 years) were interviewed. The majority of respondents (70.4%) were from Harari region, working in hospitals (78.2%), and were males (51%). In addition, more than three fourth (77.7%) had attended undergraduate degrees. While about 56.3% were nurses or midwives as compared to other professions. The average working experience of the health professionals was 5.8 years (Table 1).

### 3.2. Utilization of EMR among health professionals

A total of 363 (88.1%) of respondents reported being able to use a computer. However, among computer users, only 330 (80.1%) had access to a computer within their facility. More than half, 211 (51.2%), use it for data recording, followed by report generating and reading. The majority, 279 (67.7%), reported that they use EMR in their facility currently (54%) use it on a daily basis. Out of these, 178 (43.2%), 103 (25%), 82 (20%), and 74 (18%) use the EMR system for report generating, report sending, data retrieval, and data analysis purposes, respectively (Table 2).

Almost two-thirds, 272 (66%) of respondents reported that they prefer EMR instead of paper-based system as it saves time (51%), stores more data (33.7%), easy to access data (30.6%), easy to write a report (22.1%), and increase data quality (30.3%). At the same time, 75% reported that the EMR system is not difficult to use, and 73.4% perceive that it does not need advanced computer skills (73.4%). In addition, only 106 (26%) had even taken any EMR-related training (Table 2).

### 3.3. Knowledge and attitude towards EMR

**Figure 2:**
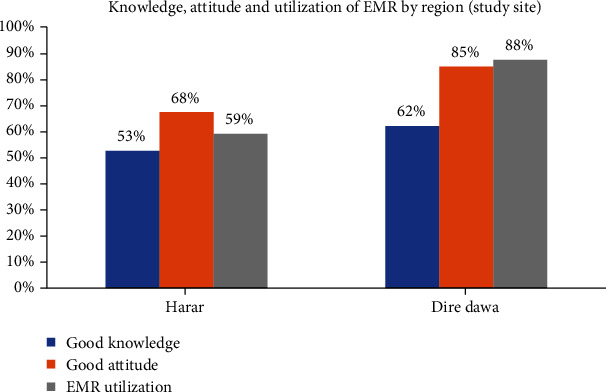
Knowledge, attitude, and utilization of EMR by region (study sites).

With subgroup analysis, about 52.8%, 67.6%, and 59.3% had good knowledge, attitude, and use of EMR, respectively, from the Harari region. While higher proportions, 62.3%, 85.2%, and 87.7% had good knowledge, attitude, and use of EMR, respectively, from Dire Dawa (Figure 2).

The majority of respondents are aware of the potential functions of EMR in handling patient data, reducing errors, and improving the quality of care. More than three-fourths of respondents agree that EMR improves timely patient care decisions, needs training, reduces medical errors, and can be potentially acceptable by users. About 352 (85.2%) of health professionals showed positive willingness to use EMR in the future (Tables 3 and 4).

The Kolmogorov-Smirnov test showed that the overall knowledge and attitude score violated the assumptions of normal distribution (*P* value was less than 0.001). Thus, the mean was used as cut-off point to classify the knowledge and attitude levels into good and poor (those who scored the mean and above were considered as having good and poor knowledge and attitude, respectively). The mean knowledge and attitude scores were 12.2 (±3.3) and 12.8 (±3.7), which were scored out of 15 knowledge measuring questions. A total of 229 (55.6%) and 300 (72.8%) of health professionals had a good knowledge and favorable attitude towards EMR utilization.

About 227 (55.1%) of respondents mentioned that there is a person assigned in the facility to facilitate EMR within the institution. The perceived main roles of the assigned person were to manage the EMR system (38%), conduct EMR (15%), give training, and generating overall report (13.3%) were the perceived roles of the assigned person. Only 68.3% of respondents responded affirmatively to the presence of facility management support for EMR. A total of 116 (28.2%) state that there is a regular meeting regarding EMR implementation within the facility. More than half (52.2%) state that the generator was the first alternative in the presence of power interruption.

### 3.4. Factors associated with utilization of EMR

Under bivariate analysis, sex, age, work experience knowledge, attitude on EMR, and other factors were associated with EMR utilization. Majority of those who currently use EMR were males (COR = 1.42; 95 CI: 0.93-2.14), work in health center (1.75; 95 CI = 1.02 − 3.01), and with specialty (COR = 2.4; 95% CI: 0.6-9.4). Health professionals with higher age, above 35 years (COR = 5.2; 95% CI; 2.3-11.8), and work experience, above five years (COR = 2.9; 95% CI: 1.7-5.0), were two and five times more likely to use EMR. Those with EMR training were more likely to use EMR than without training (COR = 5.14; 95% CI; 2.7-9.8). Furthermore, respondents with good knowledge (COR = 2.24; 95% CI; 1.42-3.42) and a good attitude (COR = 2.22, 95% CI; 1.42-3.49) were more likely to use and practice EMR with their facility (Table 5).

After controlling for confounding variables, sex, educational status, age, work experience, knowledge, attitude, and having EMR training were important factors associated with EMR utilization. Hosmer and Lemeshow's goodness of fit showed a good measure of model fitness (*χ*^2^ = 4.1, df = 8, and *P* value = 0.728) which fails to reject the null hypothesis that the model is fit. Health professionals with more than five years' experience had two times higher odds of using EMR (AOR = 2.22; 95% CI; 1.12-4.42) than early-career workers (0-2 years of experience). When compared to those who had not received EMR training, those who had previously received EMR training were significantly more likely to use EMR (AOR = 5.88; 95% CI; 2.93-11.88). In addition, having good knowledge (AOR = 1.52; 95% CI; 0.92-1.5) and a good attitude on EMR system (AOR = 2.4; 95% CI; 1.35-4.31) had 50% and 140% more odds to use EMR in their facility (Table 5).

With the assumption that the predictors of EMR use are affected by training on EMR and computer literacy. Further stratified analysis based on these factors was done. Having a good attitude, good knowledge, and work experience were significantly associated with EMR use with and without training on EMR. The model fitness was also good for both with a *P* value for the Hosmer-Lemeshow's goodness of fit test of model fitness above 5%. However, for those who are computer illiterate, knowledge, attitude, and other variables were not significant predictors. At the same time, training of individuals without computer literacy did not affect EMR use (*P* value = 0.99). On the contrary, training for those with computer literacy was shown to reduce the actual EMR use (AOR = 0.18; *P* value = 0.0001) (Table 5).

## 4. Discussion

The findings of this study showed that 67.7% of respondents used EMR, while 54% and 34% preferred an EMR system over a paper-based one, and used it on a daily basis. A shred of evidence shows that health professionals consider EMR as an easier and more effective way of handling patient records for better care [15]. However, there are so many factors that can slow down its adoption and use by health professionals. In this study, a relatively higher level of utilization was observed. It might be due to the respondent's tendency to respond in a positive way (social desirability bias or overreporting) or their actual practice. However, some facilities in the study area do not have established and functional EMR systems in all units, and others have not implemented them at the necessary scale. Findings from [25] showed that health professionals' EMR utilization decreased from 71.6% to 35% immediately at the start of EMR and three years later.

Results from northern Ethiopia [39] also showed that a comparable level of readiness to implement EMR within their facility (54.1%), and about 71% use EMR from another evidence [30]. In comparison to this, respondents also had good knowledge (74.3%) and attitude (54.6%), which might make this level of acceptability and implementation. In this study, about 55.6% and 75.6% had good knowledge and attitude towards EMR, which shows the EMR knowledge and understanding of health professionals may not be satisfactory, which ultimately results in low adoption and utilization. It may also be partly due to the fact that only 26% of respondents had EMR training, which may hinder its adoption and utilization. Even if there is training, the presence of an organized organizational support system and management is essential [7, 42, 43]. Since EMR implementation requires strong organizational commitment and support through training, resource allocation, and creating a conducive environment. This urges the need for a well-established mechanism system for strengthening and revolutionizing the sector is crucial.

Since EMR is an efficient strategy to improve the quality of patient data, missing data (*P* value less than 0.01), and overall patient health care [33], there is a need to upgrade acceptance by health care providers. This appreciates the presence of barriers and obstacles from health care providers and some organizational related factors that play an important role in the adoption and implementation of EMR at a large scale. It emphasizes the need of regional and local officials to create an enabling environment in health care institutions that is conducive for EMR implementation [19, 42].

Results from Ayder hospital showed that almost all (95%) of the units within the hospital use EMR systems, and 92% of the units are organized with functional and well-established EMR systems [17]. The use of EMR systems among health professionals in western Oromia was found to be 42% [41]. In addition, about 70.8% of health care providers from northern Ethiopia use the EMR service for service delivery [30]. This might be due to the fact that the EMR system is not well-established in a functional way in outpatient and inpatient wards, laboratory services, and other core units within hospitals for better implementation at the health professional level.

It may be partly due to some features of the system that hinder utilization, which demand an upgraded open source HER system that is more user-friendly with improved features [2]. This may decrease the job burden on health professionals where the caseload and number of health professionals are not well balanced, in the case of referral hospitals. Others complain that EMR for healthcare providers is time-consuming, adds a burden, and keeps them busy [27, 35]. Since better quality service and patient care are the ultimate goals of health facilities, there should be a way of improving EMR utilization among health workers should be improved.

Health professionals with more than five years of experience had two times higher odds of using EMR (AOR = 2.22; 95% CI; 1.12-4.42) than early-career workers (0-2 years of experience). Another study also showed that experienced health workers had a better utilization of EMR (AOR = 1.81; 95% CI: 1.04-3.16). Furthermore, the study also showed that professionals aged less than 30 years were more likely to use EMR [30]. However, experienced healthcare providers usually have skills gained during training, work stays, and other occasions that equip them with better skills and motivations than fresh graduates. Thus, it emphasizes the need to focus on fresh graduates for training and capacity building on EMR.

In addition, those with computer literacy and who have access to computers were more likely to use EMR. Similar studies showed that previous computer skills and access to computers were positively related to the use of EMR [44]. Health professionals with computer skills were two times more likely to use EMR services (AOR = 1.74; 95% CI: 1.16-2.85) [30]. It emphasizes the need to upgrade the computer skills of providers for better use of EMR through various hands-on training. Besides that, the other important point to consider is the availability of functional computers within each unit. However, the effect of training among specialist professionals was found to be negative, which might be due to the double burden of handling client information on paper-based EMR, which creates an additional burden and hinders their EMR utilization.

Moreover, having good knowledge (AOR = 1.52; 95% CI; 0.92-1.5) and a good attitude towards the EMR system (AOR = 2.4; 95% CI; 1.35-4.31) were positively associated with EMR utilization. In this study, about half of the respondents were knowledgeable about EMR, which may hinder its use. Another study also identified that lack of skills, knowledge, and motivation to use EMR were the main barriers for EMR utilization [7].

Health professionals who received EMR training were more likely to use EMR (AOR = 5.88; 95% CI; 2.93-11.88) than those who did not receive training. It is a fact that effective training and refreshment training are means of acquiring and increasing skills related to EMR and improving confidence in its use. This will allow them to refresh their knowledge and skills related to the core functions of the system. Evidence also emphasized the need for continuous training of health care providers after the start of EMR [45] coupled with a continuous support program. Even beyond this, the way of training should be shifted to an e-learning approach [46] to address the vast majority in resource-limited settings. The implementation of effective and targeted training coupled with attitude change within health care providers is crucial for improved utilization of EMR [47]. Trainees should also be selected from each unit/department rather than train HMIS focal people repeatedly. In addition, preparing in-service training will be a better option to improve the capacity and skills of staff.

This study tried to point out EMR utilization and its associated factors in this situation. Thus, the results of this study should be viewed in light of some limitations. Among these, the issue of social desirability bias (overreporting or underreporting) could not be avoided. In addition, this study did not assess the organizational readiness and overall situation in detail, which might have affected the utilization level. Otherwise, this study is a valuable input for the Harari region and Dire Dawa health bureau for evidence-based improvement of the EMR system in each facility.

## 5. Conclusion and Recommendations

The utilization of EMR was found to be optimal, and age, work experience, knowledge, attitude, and training were associated with use of EMR in their facility.

Generally, based on the findings of this study, Regional Health Bureaus are in collaboration with hospitals and health centers to strengthen and support the EMR in their facilities. There should be a continuous, targeted, and effective EMR training, and refreshment training once EMR is established. Above all, there should be measures to improve the skills and attitudes of healthcare providers towards the benefits of the implementation of EMR.

## Figures and Tables

**Table 1:** Basic characteristics of health professionals from study on utilization of EMR in Eastern Ethiopia, 2019.

Variables		Frequency	Percent
Study site	Harari	290	70.4
	Dire Dawa	122	29.6

Sex	Male	210	51.0
	Female	202	49.0

Type of facility	Hospital	322	78.2
	Health center	90	21.8

Educational level	Diploma	61	14.8
	Bsc	320	77.7
	MSc/MPH	17	4.1
	Specialty	14	3.4

Professional category	General practitioner/specialty	52	12.6
	Health offices	35	8.5
	Nurse/midwife	232	56.3
	Medical laboratory	19	4.6
	Pharmacist	39	9.5
	Others^∗^	35	8.5

^∗^Refers to health informatics and environmental health.

**Table 2:** Patterns of EMR use among health professionals in Eastern Ethiopia.

Variables		Frequency	Percent
Able to use computer (*n* = 412)	Can use	363	88.1
	Cannot use	49	11.9

Access to computers (*n* = 412)	Yes	330	80.1
	No	82	19.9

For what purpose do you use computers? (*n* = 330)	Data recording	211	51.2
	Report generating	46	11.2
	Reading	62	15.0
	Video accessing	11	2.6

Do you currently use EMR in your facility? (*n* = 412)	Yes	279	67.7
	No	133	32.3

How often do you use EMR? (*n* = 279)	Daily	223	54.1
	Three times week	24	5.8
	Once a week	19	4.6
	I do not remember	13	3.2

Report generating (*n* = 279)	Yes	178	43.2
	No	101	24.5

Report sending (*n* = 279)	Yes	103	25.0
	No	176	42.7

Data retrieving (*n* = 279)	Yes	82	19.9
	No	197	47.8
Data analysis (*n* = 279)	Yes	74	18.0
	No	205	49.8

**Table 3:** Knowledge of health professionals on EMR.

Knowledge parameters (*n* = 408) EMR system allows to ……		Frequency	Percentage
Review patient problem	Yes	351	86
	No	57	14
Seek out specific advice	Yes	344	84.3
	No	64	15.7
Follow patient results	Yes	334	81.9
	No	74	18.9
Enter daily notes	Yes	318	78
	No	90	22
Order laboratory	Yes	359	88
	No	49	12
Obtain test result	Yes	352	86.3
	No	56	13.7
Report patient status	Yes	352	72.8
	No	56	27.2
Write prescription	Yes	297	74.5
	No	111	25.5
Had role-based interface	Yes	328	80.4
	No	80	19.6
Had registered codes	Yes	309	75.7
	No	99	24.3
Improve quality	Yes	354	87.2
	No	52	22.8
Improve data handling	Yes	348	85.7
	No	58	14.3
Reduce medical error	Yes	332	81.8
	No	74	18.1
Perform clinical decision	Yes	306	75.4
	No	100	24.6
Facilitate transparency	Yes	322	79.3
	No	84	20.7

**Table 4:** Attitudes of health professionals towards use of EMR.

What you feel about, EMR allows….		Frequency	Percentage
Easy to enter data	Disagree	40	9.7
	Agree	368	89.3
Improve service quality	Disagree	54	13.1
	Agree	354	85.9
Saves cost	Disagree	82	19.9
	Agree	326	79.1
Saves time	Disagree	42	10.2
	Agree	366	88.8
Increase patient satisfaction	Disagree	62	15.0
	Agree	346	84.0
Increase health of patient	Disagree	64	15.5
	Agree	344	83.5
Support information	Disagree	50	12.1
	Agree	358	86.9
Support clinical decision	Disagree	42	10.2
	Agree	366	88.8
Increase timely decision	Disagree	57	13.8
	Agree	351	85.2
Needs training	Disagree	54	13.1
	Agree	354	85.9
Reduce medical error	Disagree	85	20.6
	Agree	323	78.4
Acceptable by professional	Disagree	63	15.3
	Agree	345	83.7
Willingness to use EMR	No	56	13.6
	Yes	352	85.4

**Table 5:** Factors associated with utilization of EMR (both crude and adjusted measure of association) in eastern Ethiopia, 2019.

Associated factors		EMR use		COR with 95% CI	*P* value	AOR	*P* value
		Yes	No				
Sex	Male	150	60	1.42 (0.93-2.14)	0.101	1.06 (0.66-1.70)	0.815
	Female	129	73	1		1	

Type of facility	Health center	69	21	1.75 (1.02-3.01)	0.042		
	Hospital	210	112	1			

Age category in years	20-24	36	41	1		1	
	25-29	152	64	2.71 (1.59-4.62)	0.0001	1.86 (1.01-3.42)	0.046
	30-34	45	18	2.85 (1.41-5.77)	0.004	1.33 (0.56-3.17)	0.514
	≥35	46	10	5.24 (2.31-11.9)	0.0001	2.54 (0.96-6.70)	0.059

Educational level	Diploma	37	24	1		1	
	BSC	219	101	1.41 (0.80-2.48)	0.237	1.35 (0.70-2.60)	0.377
	MSC/MPH	12	5	1.56 (0.49-4.98)	0.456	1.59 (0.44-5.76)	0.474
	Specialty	11	3	2.38 (0.60-9.42)	0.217	1.59 (0.34-7.40)	0.552

Work experience	0-2 years	61	53	1		1	
	3-5 years	103	46	1.95 (1.17-3.23)	0.010	1.59 (0.89-2.83)	0.117
	>5 years	115	34	2.94 (1.73-4.99)	0.0001	2.22 (1.12-4.42)	0.023

Computer literacy	Can use	274	89	27.1 (10.4-70.4)	0.0001		
	Cannot use	5	44	1			

Access computer	Yes	269	61	31.75 (15.5-65)	0.0001		
	No	10	72	1			

Prefer EMR	Yes	255	17	37.50 (19-74)	0.0001		
	No	24	60	1			

EMR training	Yes	94	12	5.14 (2.7 - 9.8)	0.0001	5.88 (2.93-11.78)	0.0001
	No	183	120	1		1	

Knowledge level	Good	173	56	2.24 (1.47-3.42)	0.0001	1.52 (0.92-2.51)	0.105
	Poor	106	77	1		1	

Attitude level	Good	218	82	2.22 (1.42-3.49)	0.001	2.41 (1.35-4.31)	0.003
	Poor	61	51	1		1	

## Data Availability

All relevant data are within the manuscript and its supporting information files.

## Abbreviations

A/COR: Adjusted/crude odd ratio
BSC: Bachelor of science
CI: Confidence interval
EMR: Electronic medical record
ART: Ante retroviral treatment
HIV/AIDS: Human immune virus/acquired immune deficiency syndrome
PLWHA: People living with HIV/AIDS.
